# Cross-sectional biomonitoring of urinary deoxynivalenol, T-2 and HT-2 toxins, and zearalenone in Japanese adults

**DOI:** 10.1265/ehpm.24-00245

**Published:** 2025-03-20

**Authors:** Toshiki Tajima, Tomohiko Isobe, Isao Saito, Takaaki Kondo, Koji Suzuki, Ryosuke Fujii, Yoshiki Tsuboi, Yoshiko Sugita-Konishi, Jun Ueyama

**Affiliations:** 1 Department of Biomolecular Sciences, Field of Omics Health Sciences, Nagoya University Graduate School of Medicine, 1-1-20 Daiko-minami, Higashi-ku, Nagoya 461-8673, Japan; 2 Health and Environmental Risk Division, National Institute for Environmental Studies, 16-2 Onogawa, Tsukuba 305-8506, Japan; 3 Department of Preventive Medical Sciences, Fujita Health University School of Medical Sciences, 1-98 Dengakugakubo, Kutsukake-cho, Toyoake 470-1192, Japan; 4 Faculty of Applied Biosciences, Tokyo University of Agriculture, 1-1-1 Sakuragaoka, Setagaya-ku, Tokyo 156-8502, Japan

**Keywords:** Mycotoxin, Human biomonitoring, Liquid chromatography–tandem mass spectrometry, Japanese adults, Urine

## Abstract

**Background:**

Among the more than 300 mycotoxins that are known to have toxic effects on animals and humans, *Fusarium* toxins deoxynivalenol (DON), T-2 and HT-2 toxins (T2/HT2), and zearalenone (ZEN) are frequently detected in domestic agricultural products. This study aimed to assess DON, T2/HT2, and ZEN exposure in Japanese adults by measuring urinary mycotoxins, observing their distributions, and making comparisons with data from other countries.

**Methods:**

A total of 201 individuals participated in the study. Twenty-four-hour urine samples were collected from young adults (34 men and 35 women) in the Tokai region (urban area) and spot urine samples were collected from middle-aged and elderly adults (64 men and 68 women) in the Donan area of Hokkaido Prefecture (rural area). Urinary DON, T2/HT2, and ZEN levels were measured using a validated liquid chromatography–tandem mass spectrometry method.

**Results:**

For DON, T2/HT2, and ZEN, the detection frequencies above the limit of detection (LOD) level (0.15, 0.13, and 0.01 µg/L, respectively) in all the samples were 53%, 26%, and 71%, respectively. The median concentrations (95^th^ percentile) of urinary DON, HT2, and ZEN were 0.19 (3.93), <LOD (0.55), and 0.02 (0.12) µg/L, respectively. Although at least one of the investigated mycotoxins was detected in the urine of 86% of study participants, the concentrations were similar to or lower than those found in other countries (from 19 reports within the past decade). Moreover, the probable daily intake (PDI) values in the present study were lower than the provisional maximum tolerable daily intake levels. The urinary mycotoxin levels did not significantly differ with respect to sex, age, or occupation.

**Conclusions:**

This study represents the first comprehensive exposure assessment for DON, T2/HT2, and ZEN in Japanese adults using human biomonitoring methods. These data provide valuable information for a better understanding of mycotoxin exposure in Japan.

**Supplementary information:**

The online version contains supplementary material available at https://doi.org/10.1265/ehpm.24-00245.

## Background

Mycotoxins are toxic secondary metabolites produced by fungi belonging to the genera *Aspergillus*, *Penicillium*, *Fusarium*, and *Alternaria* [1]. Mycotoxin production is influenced by environmental factors such as temperature and moisture. Therefore, mycotoxin contamination of agricultural products is difficult to prevent during harvest, transportation, and storage [2, 3]. Furthermore, owing to their stability, mycotoxins are difficult to remove through food processing or cooking [4]. Mycotoxins are potentially harmful to human health [5], and the most common route of mycotoxin exposure is the ingestion of contaminated food [6]. Human exposure to mycotoxins can also occur in the environment, including in occupational settings [7, 8].

*Fusarium* species are of particular concern because they produce mycotoxins that affect health, including deoxynivalenol (DON), T-2 and HT-2 toxins (T2/HT2), and zearalenone (ZEN) [9] (Fig. S1). Geographical differences exist in the prevalence of these mycotoxins in foods, such as wheat and barley grains [10]. To avoid the adverse effects of mycotoxins on human health, the World Health Organization (WHO) and agencies in various countries have established regulations to control mycotoxin-contaminated food and estimate the risk of mycotoxins. For instance, the Joint FAO/WHO Expert Committee on Food Additives (JECFA) and Food Safety Commission of Japan (FSCJ) set a provisional maximum tolerable daily intake (PMTDI) of 1 µg/kg bw/day for DON, and JECFA established a tolerable daily intake (TDI) of 25 ng/kg bw/day for T2, HT2, and diacetoxyscirpenol combined [11–13]. The TDI for ZEN of 0.25 µg/kg bw/day established by the European Food Safety Authority (EFSA) in 2016 is based on estrogenicity in pigs [14]. By comparing the TDI with estimated intake levels of DON, a probabilistic risk assessment of mycotoxin exposure was conducted using Monte Carlo simulations based on mycotoxin concentrations and food consumption levels [15]. However, more comprehensive risk assessments require the determination of individual rather than population exposure levels and clarification of the association between exposure and health effects through epidemiological studies.

The human biomonitoring (HBM) approach provides the information on chemical exposure by determining chemicals or their metabolites in biological specimens [15, 16]. Specifically, the HBM is more effective than the food assessment approach for estimating an individual’s level of exposure to specific mycotoxins, particularly when contamination status is difficult to predict [15]. Thus, HBM has been recognized as a valuable method for assessing exposure levels to various mycotoxins [8, 17], and comparable data related to mycotoxin exposure have been accumulated worldwide, as follows: Europe [18], North America [19], South America [20], Africa [21], and Asia [22]. The first urinary DON assay in a Japanese population was conducted by Xia et al. [23]. However, the number of participants was small, and a single mycotoxin was targeted. For the Japanese population, HBM data related to exposure to multiple mycotoxins remains scarce. Such information is necessary to clarify differences in mycotoxin exposure in terms of sex, age, and occupation in Japan. Given differences in race, environment, and diet, comparing mycotoxin exposure levels in Japan and other countries is also important.

This study aimed to clarify the demographic characteristics, such as sex, age, and occupation, that influence *Fusarium* toxins (DON, T2/HT2, and ZEN) exposure levels in Japanese adults using urinary analysis.

## Methods

### Chemicals and reagents

Mycotoxin mixture solution 4, which is a standard reagent containing DON (10.0 µg/mL), T2 (10.1 µg/mL), HT2 (10.1 µg/mL), and ZEN (10.0 µg/mL), was purchased from Biopure (Tulln, Austria). As an internal standard (IS), acetamiprid-*d*_3_ (10 µg/mL) was obtained from Hayashi Pure Chemical Ind., Ltd. (Osaka, Japan). Ultrapure water (LC-MS grade), methanol (LC-MS grade; for mobile phase use), acetonitrile (LC-MS grade), 1 mol/L ammonium acetate solution (LC-MS grade), and sodium chloride were purchased from FUJIFILM Wako Pure Chemical Co. (Osaka, Japan). β-Glucuronidase from *Helix pomatia* (136,400 units/mL) was obtained from Roche Diagnostics (Mannheim, Germany). Water for sample preparation procedures was distilled and deionized to 18 MΩ cm using a Milli-Q system (Millipore, Burlington, MA, USA). A solid-phase extraction (SPE) column (ISOLUTE^®^ Multi-Clean 500 mg/3 mL column; Biotage, Uppsala, Sweden) was used for mycotoxin extraction from urine samples.

### Pooled urine samples and standard solutions

Pooled urine was collected from six healthy volunteers (three males and three females) and used for all optimization studies to determine urinary mycotoxins, matrix-matched calibration curves, and validation assays. Mycotoxin standards and the acetamiprid-*d*_3_ IS were diluted with acetonitrile to prepare working reference solutions at the designated concentrations. The mycotoxin solution was stored at 4 °C, and the acetamiprid-*d*_3_ solution was stored at −20 °C in the dark without a freeze–thaw cycle.

### Sample preparation procedure

**Fig. 1:**
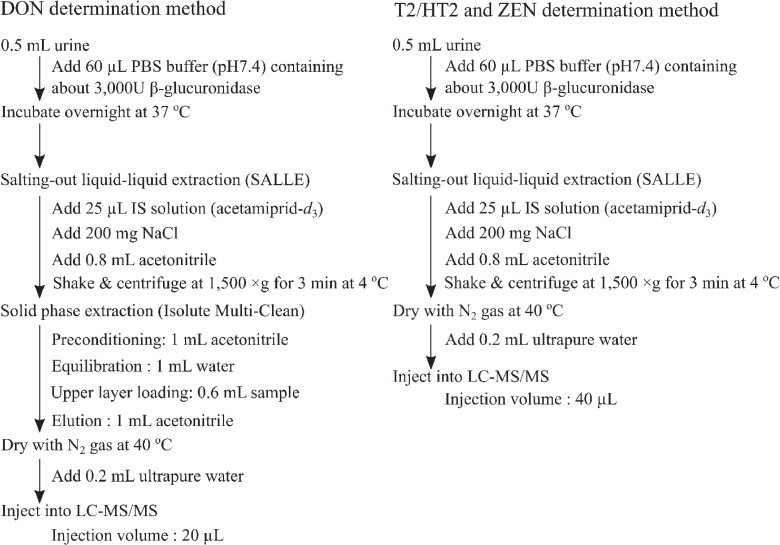
Analytical methods for determination of urinary deoxynivalenol (DON), T-2 and HT-2 toxins (T2/HT2), and zearalenone (ZEN).

A flowchart of the sample preparation procedure for urinary mycotoxin determination is shown in Fig. 1. To deconjugate DON, HT2, and ZEN, 60 µL of PBS buffer (pH 7.4) containing 3,000 U of β-glucuronidase (corresponding to approximately 6,000 units/mL urine) was added to 0.5 mL of urine. The samples were then incubated for 18 h at 37 °C. During the deconjugation procedure, T2 was converted to HT2; therefore, the sum of the T2 and HT2 concentrations (T2/HT2) was used hereafter.

Salting-out-assisted liquid–liquid extraction (SALLE) was used to prepare the urine samples for the determination of T2/HT2 and ZEN. Briefly, after enzymatic hydrolysis, 25 µL of IS solution (200 µg/L acetamiprid-*d*_3_), 0.3 g of sodium chloride, and 0.8 mL of acetonitrile were added. The mixture was vortexed and centrifugated at 1,500 × g for 3 min at 4 °C. The upper layer was transferred to a polypropylene tube and dried on a heat block at 40 °C using a gentle nitrogen stream. Finally, the residue was dissolved in 200 µL of water.

Urinary DON was determined using SALLE combined with SPE. Briefly, the Multi-Clean SPE column was preconditioned by washing with 1 mL of methanol, followed by conditioning with 1 mL of water. The upper layer obtained following the SALLE procedure (0.6 mL) was loaded onto a preconditioned Multi-Clean SPE column. The target compounds were eluted with 1 mL of methanol. The eluted fraction was transferred to a polypropylene tube and dried on a heat block at 40 °C using a gentle nitrogen stream. Finally, the residue was dissolved in 200 µL of water.

### Chromatography and mass spectrometry

To quantify DON, T2/HT2, and ZEN, the prepared urine samples were analyzed using liquid chromatography–tandem mass spectrometry (LC-MS/MS). LC-MS/MS analysis was performed using an Agilent 1290 Infinity II Bio LC system coupled with an Agilent Ultivo triple quadrupole mass spectrometer (Agilent Technologies, Inc., Santa Clara, CA, USA). The LC operating conditions were as follows: column, Raptor FluoroPhenyl LC column (100 × 2.1 mm, 2.7 µm; Restek, Bellefonte, PA, USA) fitted with a Raptor FluoroPhenyl EXP guard column cartridge (5 × 2.1 mm, 2.7 µm; Restek, Bellefonte, PA, USA); mobile phase A, water containing 10 mmol/L ammonium acetate; mobile phase B, methanol; gradient conditions for of mobile phase B, 0% (0–1 min), 20% (1.01 min), 90% (1.01–7 min), 90% (7–9 min), 0% (9.01 min), and 0% (9.01–13 min); total flow rate, 0.4 mL/min; total run time per sample, 13 min; injection volume for SALLE/SPE method: 20 µL; and injection volume for SALLE method, 40 µL.

The MS/MS instrument was operated with an Agilent jet stream source in positive and negative ion modes with multiple reaction monitoring (MRM). The nebulizer gas pressure, source temperature, and gas flow rate were 45 psi, 300 °C, and 10 L/min, respectively. The sheath gas temperature and gas flow rate were 250 °C and 12 L/min, respectively. The capillary voltage was 4,000 V, and high-purity nitrogen gas was used in the collision cell. Table S1 shows the optimized MRM parameters and retention times for the mycotoxins and IS. Chromatographic and mass spectrometry data were collected using a Mass Hunter Software Workstation (Agilent Technologies, Inc., Santa Clara, CA, USA). MRM chromatograms of pooled urine spiked with mycotoxins are shown in Fig. S2.

### Assay validation

The bioanalytical method was validated in terms of extraction recovery, precision, limit of detection (LOD), and lower limit of quantification (LLOQ). The assay validation data are presented in Table 1.

**Table 1:** Recovery rate, precision, LOD, and LLOQ data for the analytical procedure.

	**Concentration** **(µg/L)**	***n***	**DON**	**T2/HT2**	**ZEN**
Absolute recovery rate (%)					
	0.75	3	60	114	68
	5	3	67	128	73
	30	3	63	125	66
Within-run precision (%RSD)					
	0.75	6	8.3	3.9	8.1
	5	6	3.8	3.1	6.1
	30	6	11.9	1.0	7.9
Between-run precision (%RSD)					
	0.75	2	12.7	5.9	1.3
	5	2	8.7	2.7	7.1
	30	2	8.8	6.7	12.4

LOD (µg/L, S/N = 3)			0.15	0.13	0.01
LLOQ (µg/L)			0.50	0.45	0.03

*n*: number of observations; RSD: relative standard deviation; LOD: limit of detection; LLOQ: lower limit of quantification; S/N: signal-to-noise ratio

Absolute recovery rates were estimated at three concentration levels: 0.75, 5, and 30 µg/L (*n* = 3). Recovery rates were calculated by comparing the peak areas determined using the following two procedures. Samples in the first set were spiked with mycotoxins prior to sample preparation. Samples in the second set were spiked immediately before LC-MS/MS analysis.

The within-run precision was evaluated by analyzing pooled urine spiked with mycotoxins at concentrations of 0.75, 5, and 30 µg/L (*n* = 6). The between-run precision was evaluated at concentrations of 0.75, 5, and 30 µg/L (*n* = 2) for five consecutive days.

The LOD and LLOQ were calculated by assuming signal-to-noise (S/N) ratios of 3 and 10, respectively. The LLOQ of within-run precision was defined as less than 20% (relative standard deviation, RSD).

### Study participants and design

Table 2 presents the basic characteristics of the study participants. The two groups were selected based on three criteria. First, residence in different regions; second, differences in their age; third, feasibility of collecting urine samples at limited cost and effort. The study participants were university students living in the Tokai area, Japan (hereafter referred to as Tokai) and middle-aged and elderly adults living in the Donan area, Hokkaido, Japan (hereafter referred to as Hokkaido) who attended a health checkup program. The Tokai participants consisted of 69 university students (34 males and 35 females) with an age range of 20–29 years, and 69 24-h urine samples were collected from 2020 to 2021. The Hokkaido participants consisted of 132 adults (64 males and 68 females) with an age range of 40–87 years, and 132 spot urine samples were collected in 2023. A self-administered questionnaire was used to collect demographic data such as sex, age, and occupation. Although sex-related differences in urinary mycotoxins were studied in both groups, age- and occupation-related differences were only studied in the Hokkaido group because of the limited range of ages and occupations in the Tokai group. The other characteristics of the participants obtained from self-administered questionnaire were summarized in Table S2. The Ethical Review Committees of Nagoya University Graduate School of Medicine approved the study protocol (2020-0187 and 2023-0385).

**Table 2:** Characteristics of Tokai and Hokkaido participants.

**Characteristic**	**Tokai**	**Hokkaido**
Sample collection (month/year)	10/2020–7/2021	8/2023
Sample type	24-h urine	Spot urine
Number of participants (male, female)	69 (34, 35)	132 (64, 68)
Age (years)	22 ± 2 (20–29)	65 ± 10 (40–87)
BMI (kg/m^2^)	20.9 ± 2.9 (16.9–38.3)	24.0 ± 3.8 (14.2–35.3)
Creatinine concentration (g/L)	1.21 ± 0.53 (0.28–2.75)	1.35 ± 0.81 (0.12–4.87)

The age, BMI, and creatinine concentration values are presented as mean ± SD (min-max).

### Measurement of urinary creatinine concentrations

For the Hokkaido participants, the creatinine concentrations in the urine samples were measured using high-performance liquid chromatography (HPLC) with UV detection according to our previous method [24]. Briefly, urine samples were diluted 20-fold with H_2_O, and standard creatinine solutions were prepared at concentrations of 2, 4, and 6 mg/dL with H_2_O in HPLC vials. Analyses were performed using an Agilent HPLC 1100 series instrument (Agilent, Inc., Santa Clara, CA, USA). The within-day and between-series precisions were 0.2% and 1.3%, respectively. For the Tokai participants, the creatinine concentrations in urine were measured by a commercial laboratory (SRL Co. Inc., Tokyo, Japan) with an enzymatic assay.

### Exposure assessment

Mycotoxin intake among participants was estimated based on the results of the urinary mycotoxin analysis. The following equation was used to assess the probable daily intake (PDI) of mycotoxin [25].

$$\text{PDI (µg/kg bw/day)}=\frac{C\text{ (µg/day)}\times 100}{W\text{ (kg)}\times \text{UER}\text{ (\%)}}$$ (1)

$$\text{PDI (µg/kg bw/day)}=\frac{C\text{ (µg/g cre)}\times 100}{W\text{ (kg)}\times \text{UER}\text{ (\%)}}$$ (2)

where *C* is the urinary concentration of the mycotoxin biomarker (µg/day or µg/g cre) and *W* is the body weight of the participant (kg). *UER*, which refers to the urinary excretion rate (%) of the corresponding mycotoxin, was calculated to be 64% for DON [26] and 9.4% for ZEN [27]. Owing to the different urine sample types, equation (1) was used for the Tokai participants (24-h urine) and equation (2) was used for the Hokkaido participants (spot urine).

### Data analysis

Deviation from the normal distribution was examined using the Kolmogorov–Smirnov test. The differences in urinary mycotoxin concentrations between the two groups (sex or geographical differences) were examined using the Mann–Whitney *U*-test, whereas those among the nine occupational groups were examined using the Kruskal–Wallis test. All statistical analyses were conducted using the JMP^®^ Pro statistical software (Version 16; SAS Institute Inc., Cary, NC, USA), and *p*-value < 0.05 was considered statistically significant. Undetectable urinary concentrations of mycotoxins were estimated as the LOD value divided by the square root of 2 to calculate the geometric mean (GM) [28].

## Results

### Mycotoxin concentrations in urine

The urinary concentrations of mycotoxins are summarized in Table 3. The units are µg/L, µg/g creatinine, and µg/day for the Tokai group, and µg/L and µg/g creatinine for the Hokkaido group. The mycotoxin detection frequencies in the urine samples, expressed as the percentage of samples with above LOD levels, were 90% for ZEN, 62% for DON, and 23% for T2/HT2 in the Tokai group and 61% for ZEN, 48% for DON, and 21% for T2/HT2 in the Hokkaido group. At least one mycotoxin was detected for 86% of all participants. In addition, the urinary concentration of DON was higher than those of T2/HT2 and ZEN. In this study, the urinary concentrations of mycotoxins are presented as the GM, median values, and percentile values because of their nonparametric distribution. In all the samples, the median (50^th^) concentrations were approximately 0.2 µg/L for DON, below the LOD (<LOD) for T2/HT2, and 0.02 µg/L for ZEN. The maximum urinary levels of DON and ZEN were 20–30 times higher than the median levels.

**Table 3:** Detection frequencies and percentiles of urinary mycotoxin concentrations.

	**Analyte**	**>LOD** **(%)**	**GM**	**Selected percentile**			**Max**

				**50^th^**	**75^th^**	**95^th^**	
Tokai (*n* = 69)							
µg/L	DON	62	0.30	0.22	0.71	3.33	4.21
	T2/HT2	23	-^a^	<LOD	<LOD	0.33	1.11
	ZEN	90	0.03	0.03	0.06	0.11	0.61

µg/g cre	DON		0.26	0.26	0.63	2.29	3.39
	T2/HT2		-	<LOD	<LOD	0.25	0.46
	ZEN		0.02	0.03	0.05	0.08	0.34

µg/day	DON		0.29	0.21	0.52	2.29	3.99
	T2/HT2		-	<LOD	<LOD	0.25	0.72
	ZEN		0.03	0.03	0.05	0.10	0.49
Hokkaido (*n* = 132)							
µg/L	DON	48	-	<LOD	1.75	4.65	14.34
	T2/HT2	27	-	<LOD	0.15	0.58	3.69
	ZEN	61	0.02	0.01	0.03	0.13	0.31

µg/g cre	DON		0.29	<LOD	1.22	5.57	17.59
	T2/HT2		-	<LOD	0.10	0.42	3.09
	ZEN		0.01	0.01	0.03	0.09	0.32

LOD: limit of detection; GM: geometric mean; cre: creatinine

^a^GM was not calculated due to the low detection rate.

### Demographic variables and urinary mycotoxin levels

**Fig. 2:**
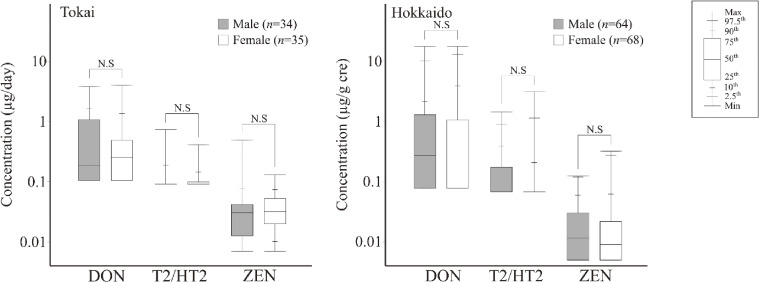
Sex differences in urinary mycotoxin concentrations in Tokai (*n* = 69) and Hokkaido (*n* = 132). “N.S” represents “not significant in Mann–Whitney *U*-test.”

**Fig. 3:**
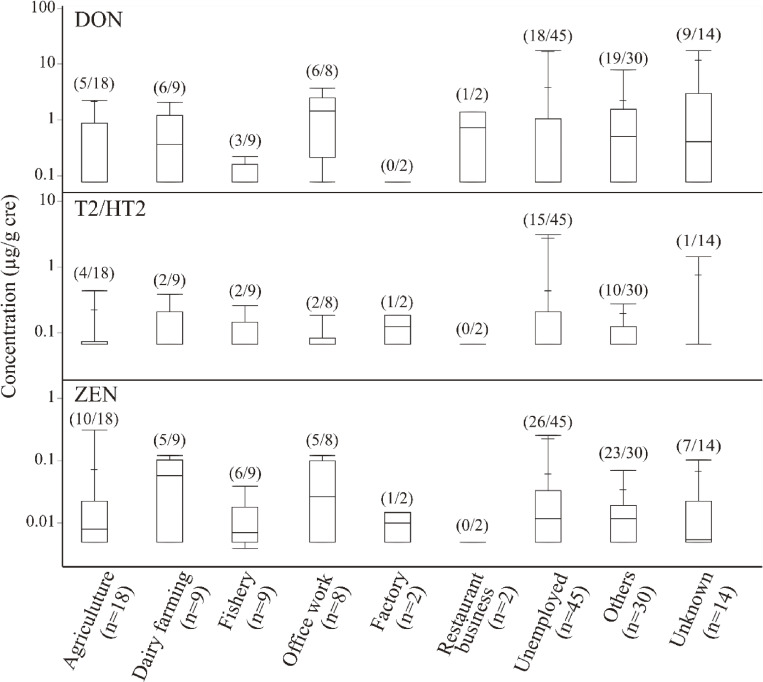
Occupational differences in urinary mycotoxin concentrations in Hokkaido (*n* = 132). The values in parentheses indicate [the number of >LOD/total number] for the corresponding parameter. For concentrations below the LOD, a value equal to the LOD divided by the square root of 2 was used in the boxplots.

Considering that different urine collection methods were used for the Tokai and Hokkaido groups, the demographic variables were studied using 24-h excretion for the Tokai participants (µg/day) and creatinine-adjusted concentrations for the Hokkaido participants (µg/g cre). The relationships between sex-specific and urinary mycotoxin concentrations are shown in Fig. 2. The urinary levels of DON, T2/HT2, and ZEN were not significantly different between males and females in either group. The relationship between occupation type and urinary mycotoxins concentrations was also examined (Fig. 3). In this analysis, nine occupations were considered. As some participants had a second job, the total count (137) exceeds the actual number of participants (*n* = 132). None of the occupations were significantly correlated with urinary mycotoxin concentrations.

### Estimated dietary mycotoxins intake and exposure assessment

As UER values for DON and ZEN are available from previous studies, PDI values could be evaluated based on the urinary concentrations of these mycotoxins. The PDI values of T2/HT2 could not be calculated because of the lack of information regarding the UER. Table 4 summarizes the calculated PDI values and the TDI or PMTDI values established by risk assessment committees. The PDI values of all participants were below the TDI or PMTDI levels. For DON, the 95th percentile PDI value was approximately 10% of the PMTDI value defined by JECFA and FSCJ. For ZEN, the 95th percentile PDI value was approximately 4–8% of the PMTDI value established by JECFA and the TDI value established by EFSA.

**Table 4:** PDI of DON and ZEN in Tokai and Hokkaido participants

**Mycotoxin**	**UER** **(%)**	**PDI ** **(µg/kg bw/day)**			***P*-value for difference^a^**	**PMTDI** **(µg/kg bw/day)**

		**GM**	**95^th^**	**Max**		
DON						
Tokai	64	0.015	0.076	0.115	*P* = 0.03	1^b^
Hokkaido		0.032	0.155	0.575		
ZEN						
Tokai	9.4	0.008	0.020	0.079	*P* < 0.01	0.5^c^, 0.25^d^
Hokkaido		0.005	0.016	0.070		

UER: urinary excretion rate; PDI: probable daily intake; PMTDI: provisional maximum tolerable daily intake; GM, geometric mean

^a^Mann–Whitney *U* test.

^b^Defined by the Joint FAO/WHO Expert Committee on Food Additives (JECFA) and Food Safety Commission Japan (FSCJ).

^c^Defined by JECFA.

^d^Defined by the European Food Safety Authority (EFSA) as tolerable daily intake (TDI).

Although the sample collection methods differed, the PDI values of the two groups were compared. The PDI values of DON in the Tokai group were statistically significantly lower than those in the Hokkaido group (*P* = 0.03). In contrast, for ZEN, the PDI values in the Hokkaido group were significantly lower than those in the Tokai group (*P* < 0.01).

## Discussion

Mycotoxin occurrence trends vary in different regions because differences in climatic conditions affect mycotoxin formation during crop growth and storage [29]. Moreover, mycotoxin exposure is related to food consumption and work or living environments. Thus, understanding the mycotoxin exposure levels in various regions is important for developing regulatory science in the county level. This study, which is the first to simultaneously assess DON, T2/HT2, and ZEN exposure levels in a large number of Japanese adults using HBM techniques, reveals two important findings regarding the characteristics of mycotoxin exposure in Japan. First, our results show that a high proportion of 24-h and spot urine samples provided by healthy adults residing in two different areas of Japan had detectable levels of the mycotoxins DON, T2/HT2, and ZEN, with at least one mycotoxin being detected in the urine of 86% of study participants. However, these concentrations are approximately the same as or lower than those previously reported in other countries. Second, despite the limitations of the data, the urinary DON, T2/HT2, and ZEN concentrations did not differ significantly according to sex, age, or occupation. However, regional differences were observed in the PDI values of DON and ZEN.

Studies reporting urinary mycotoxin concentrations among farmers and the general population within specific age ranges, such as children, pregnant women, and adults, have rapidly increased since 2015, concomitant with methodological development. Table 5, Table S3, and Table S4 summarize our biomonitoring data as well as the results of previous biomonitoring studies on DON, T2/HT2, and ZEN urinary concentrations in adults. The median values are not included for some studies because of the low detection frequency (%). In comparison with reports that provide median or 95^th^ percentile values, the urinary mycotoxin levels in the Japanese population are comparable or lower. High urinary concentrations of DON were reported in two studies from France involving farm workers [39, 40] and one study from China involving the general population [36]. According to a report by the Rapid Alert System for Food and Feed, most notifications of mycotoxin contamination concern EU-imported food products originating from China [41, 42]. No appropriate data are available for comparing the extent of DON and ZEN contamination in Japan with that in foreign countries. However, the biomonitoring results imply that the DON and ZEN exposure levels for some Japanese adults are similar to or lower than the exposure levels in other countries.

**Table 5:** DON concentrations in the present study and similar previous studies focusing on adult exposure.

**Study**	**Country**	**Age** **mean ± SD** **(min-max)**		***n***	**DF** **(%)**	**Concentration (µg/L)**		

						**50^th^**	**95^th^**	**Max**
Nonoccupational exposure								
Martins et al. [30]	Portugal	48 ± 15		94	63^a^	2.5	16.8	36.3
					30^b^	0.4^f^	5.3	9.4
Warth et al. [31]	Austria		(20–63)	27	59^b^			63
Heyndrickx et al. [15]	Belgium		(19–65)	239	37^b^	1.7^f^		129.8
Gerding et al. [32]	Germany	-		50	16^c^			2
Carballo et al. [33]	Spain		(18–65)	40	23^b^			18.7
De Ruyck et al. [34]	Europe		(45–65)	188	24^a^	0.5^f^	3.7^f^	9.1
Ali et al. [35]	Bangladesh	39 ± 11	(22–60)	62	27^b,d^			1.8
					31^b,e^			1.2
Deng et al. [36]	China	40 ± 13	(21–64)	68	100^b^	29.8		213
Huang et al. [37]	China	45 ± 18	(20–88)	227	12^b^			8.6
Xia et al. [23]	Japan		(22–25)	30	90^b^			28.5
Collins et al. [38]	Rwanda	30	(18–55)	119	19^c^	16.3		57.7

Occupational exposure								
Ndaw et al. [39]	France	-		9	100^b^	14.4		18.8
Ndaw et al. [40]	France		(19–56)	195	98^c^	14.5		154
Foerster et al. [3]	Chile	57 ± 9	(35–74)	172	55^b^	37.6		61.1
Xia et al. [22]	Pakistan	37 ± 17		292	35^c^			3.5

Present study								
Tokai	Japan	22 ± 2	(20–29)	70	63^a^	0.2	3.3	4.2
Hokkaido	Japan	65 ± 10	(40–87)	132	48^c^		4.7	14.3

DF: detection frequency above the limit of detection (LOD)

^a^24-h urine, ^b^First morning urine, ^c^Spot urine, ^d^Collected in summer, ^e^Collected in winter, ^f^75^th^ percentile

Similar to JECFA, the FSCJ set a PMTDI of 1 µg/kg bw/day for DON, which is based on the dose observed to suppresses weight gain in a 2-year chronic toxicity study in mice [43]. According to the PDI determined in the present study, the DON exposure levels of all the study participants were lower than the PMTDI (Table 4). The daily DON intake levels were previously estimated using the mean DON contamination level in food as approximately 0.13–0.17 µg/kg bw/day for all ages and 0.29–0.36 µg/kg bw/day for children (1–6 years old) [43], which exceed the PDI value determined in the present study. This discrepancy may in part be due to UER uncertainty, underrepresentation in urine samples, or inappropriate evaluation of food processing or cooking in the estimation. Although limited ZEN exposure assessments have been performed in Japan, the PDI of ZEN could be calculated in this study using the HBM technique. The obtained GM and maximum levels of 0.008–0.009 and 0.077–0.079 µg/kg bw/day, respectively, represent 1.7% and 16% of the PMTDI set by JECFA. These results suggest that the DON and ZEN exposure risks are typically well controlled in Japanese adults. Unfortunately, the PDI of T2/HT2 could not be calculated because data on the human UER of T2/HT2 is currently unavailable. T2/HT2, which are the most toxic type A trichothecenes, can affect human health through oxidative stress-mediated cytotoxicity or immune system suppression [44, 45]. Therefore, UER data on T2/HT2 is urgently needed to facilitate risk assessments using HBM. Because mycotoxin contamination of baby food has been reported [46], mycotoxin exposure in children, who are vulnerable to toxins, is of particular concern. Thus, further nationwide studies covering a wide age range, including children, are needed to monitor DON, T2/HT2, and ZEN in urine.

No sex-, age-, or occupation-related differences were observed in the urinary concentrations of DON, T2/HT2, and ZEN. Contradictory results have been reported in the literature on sex differences in urinary mycotoxins. Although some previous studies have reported no significant association between sex and DON or ZEN exposure in adults [3, 35], Turner et al. [47] found that the urinary concentration of DON in males was significantly higher than that in females based on National Diet and Nutrition Survey in the UK, even after adjusting for cereal intake.

No significant differences were found in urinary mycotoxin concentrations among workers in the present study. The low statistical power resulting from the small sample size for each occupation made detecting any trends in the DON, T2/HT2, and ZEN concentrations difficult. This is a limitation of our approach, as the sample size in the present study was nearly the maximum that we could achieve. As shown in Table 5, Table S3, and Table S4, biomonitoring methods have rarely been employed to investigate occupational mycotoxin exposure. Although very significant occupational exposure to high concentrations of mycotoxins has not been reported, Foerster et al. [3] found occupational exposure to DON among grain elevator employees using HBM. Based on surveys of DON, T2/HT2, and ZEN contamination in cow and fish feed [48, 49], farm workers may be exposed to animal feed contaminated with mycotoxins. Thus, further biomonitoring research is required to determine occupational mycotoxin exposure and prevent possible health effects in workers.

The PDI values of DON, T2/HT2, and ZEN were assessed in two Japanese regions (Tokai and South Hokkaido). Note that the urine sample types differed for these groups (24-h urine vs. spot urine). Nevertheless, the PDI values of DON in the Tokai group were significantly higher than those in the Hokkaido group, whereas the opposite trend was observed for the PDI values of ZEN.

These results may reflect regional differences in mycotoxin exposure levels. However, this finding is not conclusive because the ages of the two groups differed significantly. Limited information is currently available regarding the occurrence of mycotoxin contamination in foods across various regions. Thus, to elucidate regional differences in mycotoxin exposure, further studies are required using HBM techniques and assessments of contamination levels in food.

## Conclusions

Although at least one of the investigated mycotoxins (DON, T2/HT2, or ZEN) was detected in the urine of 86% of study participants, the concentrations were similar to or lower than those reported in other countries. Moreover, the PDI values in the present study were lower than the PMTDI or TDI levels. No significant difference was observed in the urinary concentrations of the investigated mycotoxins based on sex, age, or occupation. Further research is essential to better understand mycotoxin exposure risks, particularly focusing on diverse geographical regions and various demographic groups, including pregnant women, newborns, and toddlers.

## Abbreviations

DON: deoxynivalenol
EFSA: European Food Safety Authority
FSCJ: Food Safety Commission of Japan
GM: geometric mean
HBM: human biomonitoring
HPLC: high-performance liquid chromatography
HT2: HT-2 toxin
IS: internal standard
JECFA: Joint FAO/WHO Expert Committee on Food Additives
LC-MS/MS: liquid chromatography–tandem mass spectrometry
LLOQ: lower limit of quantification
LOD: limit of detection
MRM: multiple reaction monitoring
PDI: probable daily intake
PMTDI: provisional maximum tolerable daily intake
RSD: relative standard deviation
S/N: signal-to-noise ratio
SALLE: salting-out-assisted liquid–liquid extraction
SPE: solid-phase extraction
T2: T-2 toxin
TDI: tolerable daily intake
UER: urinary excretion rate
WHO: World Health Organization
ZEN: zearalenone

## Supplementary information

**Additional file 1:** **Table S1** Compound-specific mass spectrometer settings. **Table S2** Characteristics of the participants obtained from self-administered questionnaire. **Table S3** Total T2/HT2 concentrations in the present study and previous similar studies focusing on adult exposure. **Table S4** ZEN concentrations in the present study and previous similar studies focusing on adult exposure. **Fig. S1** Chemical structures of the mycotoxins measured in this study. **Fig. S2** MRM chromatograms of mycotoxins in pooled human urine spiked with mycotoxins at two different concentrations of mycotoxins.

## Declarations

### Availability of data and materials

Owing to the nature of this research, participants of this study did not agree for their data to be shared publicly; thus, supporting data are unavailable.
