# Supplementary material for: Cross-sectional biomonitoring of urinary deoxynivalenol, T-2 and HT-2 toxins, and zearalenone in Japanese adults
Source: Environ Health Prev Med. 2025 Mar 20;30:19. doi: 10.1265/ehpm.24-00245 (PMC11955799; doi:10.1265/ehpm.24-00245)

Supplementary Material

**Cross-sectional biomonitoring of urinary deoxynivalenol, T-2 and HT-2 toxins, and zearalenone in Japanese adults**

Toshiki Tajima, Tomohiko Isobe, Isao Saito, Takaaki Kondo, Koji Suzuki, Ryosuke Fujii, Yoshiki Tsuboi, Yoshiko Sugita-Konishi, and Jun Ueyama*

*** Correspondence:** Jun Ueyama: ueyama@met.nagoya-u.ac.jp

| Table S1 Compound-specific mass spectrometer settings. | | | | | | |
| --- | --- | --- | --- | --- | --- | --- |
| Compound | Fragmentor voltage  (V) | Collision energy (eV) | Polarity | Precursor ion  (*m/z*) | Product ion  (*m/z*) | Retention time  (min) |
| DON | 100 | 10 | positive | 297 | 249 (Q)  231 (C) | 3.2 |
| T2/HT2 | 100 | 10 | positive | 442 | 215 (Q)  323 (C) | 6.6 |
| ZEN | 150 | 30 | negative | 317 | 131 (Q)  175 (C) | 7.8 |
| Acetamiprid-*d*_3_ (IS) | 120 | 18 | positive | 227 | 126 (Q)  59 (C) | 5.5 |
| Q: quantification ion; C: confirmation ion; IS: internal standard | | | | | | |

| Table S2 Characteristics of the participants obtained from self-administered questionnaire. | | | | | | |
| --- | --- | --- | --- | --- | --- | --- |
|  | | Tokai | |  | Hokkaido | |
|  |  | n | % |  | n | % |
| Smoking status | |  |  |  |  |  |
|  | Never | 64 | 93 |  | 60 | 45 |
|  | Past smoker | 1 | 1 |  | 51 | 39 |
|  | Curren smoker | 4 | 6 |  | 21 | 16 |
| Drinking status | |  |  |  |  |  |
|  | Never | 19 | 27 |  | 59 | 45 |
|  | Past drinker | 0 | 0 |  | 3 | 2 |
|  | Current drinker | 50 | 72 |  | 70 | 53 |
| Underlying disease | |  |  |  |  |  |
|  | Allergy | 26 | 38 |  | 52 | 39 |
|  | Hypertension | 0 | 0 |  | 42 | 32 |
|  | Hyperlipidemia | 0 | 0 |  | 24 | 18 |
|  | Asthma | 0 | 0 |  | 9 | 7 |
|  | Diabetes | 1 | 1 |  | 8 | 6 |
|  | Glaucoma | 0 | 0 |  | 7 | 5 |
|  | Cataract | 0 | 0 |  | 6 | 4.5 |
|  | Thyroid disease | 0 | 0 |  | 5 | 4 |
|  | Macular degeneration | 0 | 0 |  | 4 | 3 |
|  | Liver disease | 0 | 0 |  | 3 | 2 |
|  | Gallstone disease | 0 | 0 |  | 3 | 2 |
|  | Gastric/duodenal ulcer | 0 | 0 |  | 3 | 2 |
|  | Cold sore | 0 | 0 |  | 3 | 2 |
|  | Stroke | 0 | 0 |  | 2 | 1.5 |
|  | Ischemic heart disease | 0 | 0 |  | 2 | 1.5 |
|  | Kidney disease | 0 | 0 |  | 2 | 1.5 |
|  | Cancer | 0 | 0 |  | 2 | 1.5 |
|  | Atopic dermatitis | 1 | 1 |  | 1 | 0.8 |

| **Table S3** Total T2/HT2 concentrations in the present study and previous similar studies focusing on adult exposure. | | | | | | | |
| --- | --- | --- | --- | --- | --- | --- | --- |
| Study | Country | Age  mean ± SD  (min-max) | *n* | DF  (%) | Concentration (µg/L) | | |
|  |  |  |  |  | 50^th^ | 95^th^ | Max |
| Nonoccupational exposure | | | | | | | |
| De Ruyck et al. [34] | Europe | (45–65) | 188 | 6^a^ | 0.5 | 1.1^g^ | 4.7 |
| Niknejad et al. [50]^d^ | Iran | 34 (20–46) | 10 | 10^b,e^ |  |  | 24.0 |
|  |  | 69 (50–92) | 17 | 18^b,f^ |  |  | 50.4 |
|  |  |  |  |  |  |  |  |
| Occupational exposure | | | | | | | |
| Ndaw et al. [40] | France | (19–56) | 195 | 4^c^ |  |  | 5.6 |
| Mendes et al. [51] | Algeria | 43 ± 8 | 96 | 77^b^ |  |  | 11.0 |
|  |  |  |  |  |  |  |  |
| Present study |  |  |  |  |  |  |  |
| Tokai | Japan | 22 ± 2 (20–29) | 70 | 24^a^ |  | 0.4 | 1.1 |
| Hokkaido | Japan | 65 ± 10 (40–87) | 132 | 27^c^ |  | 0.6 | 3.7 |
| DF: detection frequency above the limit of detection (LOD)  ^a^ 24-h urine, ^b^ First morning urine, ^c^ Spot urine, ^d^ GC-MS/MS analysis, ^e^ Healthy control,  ^f^ Esophageal cancer patient, ^g^ 75^th^ percentile | | | | | | | |

| **Table S4** ZEN concentrations in the present study and previous similar studies focusing on adult exposure. | | | | | | | |
| --- | --- | --- | --- | --- | --- | --- | --- |
| Study | Country | Age  mean ± SD  (min-max) | *n* | DF (%) | Concentration (µg/L) | | |
|  |  |  |  |  | 50^th^ | 95^th^ | Max |
| Nonoccupational exposure | | | | | | | |
| Martins et al. [30] | Portugal | 48 ± 15 | 94 | 48^a^ | 0.17^f^ | 2.93 | 3.98 |
|  |  |  |  | 57^b^ | 1.3 | 3.85 | 11.51 |
| Ali and Degan [52] | Germany | 37 ± 13 (16–65) | 60 | 100^b^ | 0.07 |  | 0.28 |
| Carballo et al. [33] | Spain | (18–65) | 40 | 40^b^ |  |  | 29.01 |
| De Ruyck et al. [34] | Europe | (45–65) | 188 | 3^a^ | 0.77^f^ | 2.11^f^ | 2.95 |
| Ali and Degan [53] | Bangladesh | 39 ± 11 | 62 | 100^b,d^ | 0.02 |  | 0.08 |
|  |  |  | 62 | 100^b,e^ | 0.03 |  | 0.20 |
| Li et al. [54] | China | (19–65) | 135 | 69^b^ | 0.12 |  | 1.6 |
| Huang et al. [37] | China | 45 ± 18 (20–88) | 227 | 12^b^ |  |  | 18.35 |
| Collins et al. [38] | Rwanda | 30 (18–55) | 119 | 30^b^ | 1.98 |  | 3.77 |
|  |  |  |  |  |  |  |  |
| Occupational exposure | | | | | | | |
| Ndaw et al. [39] | France | - | 9 | 67^b^ | 0.16 |  | 0.36 |
| Ndaw et al. [40] | France | (19–56) | 195 | 99^b^ | 0.13 |  | 1 |
| Mendes et al. [51] | Algeria | 43 ± 8 | 96 | 90^b^ |  |  | 126.8 |
| Xia et al. [22] | Pakistan | 37 ± 17 | 292 | 37^c^ |  |  | 0.67 |
|  |  |  |  |  |  |  |  |
| Present study |  |  |  |  |  |  |  |
| Tokai | Japan | 22 ± 2 (20–29) | 70 | 90^a^ | 0.03 | 0.22 | 0.61 |
| Hokkaido | Japan | 65 ± 10 (40–87) | 132 | 61^c^ | 0.01 | 0.13 | 0.31 |
| DF: detection frequency above the limit of detection (LOD)  ^a^ 24-h urine, ^b^ First morning urine, ^c^ Spot urine, ^d^ Collected in summer, ^e^ Collected in winter, ^f^ 75^th^ percentile | | | | | | | |

Figure Legends

**Fig. S1** Chemical structures of the mycotoxins measured in this study.

**Fig. S2** MRM chromatograms of mycotoxins in pooled human urine spiked with mycotoxins at two different concentrations of mycotoxins.

Fig. S1


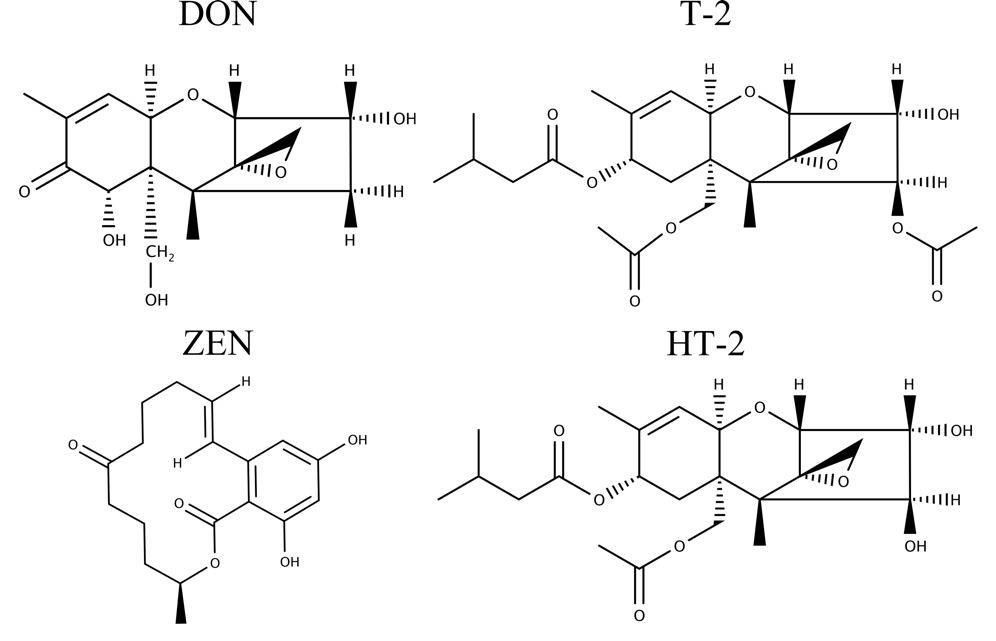


Fig. S2


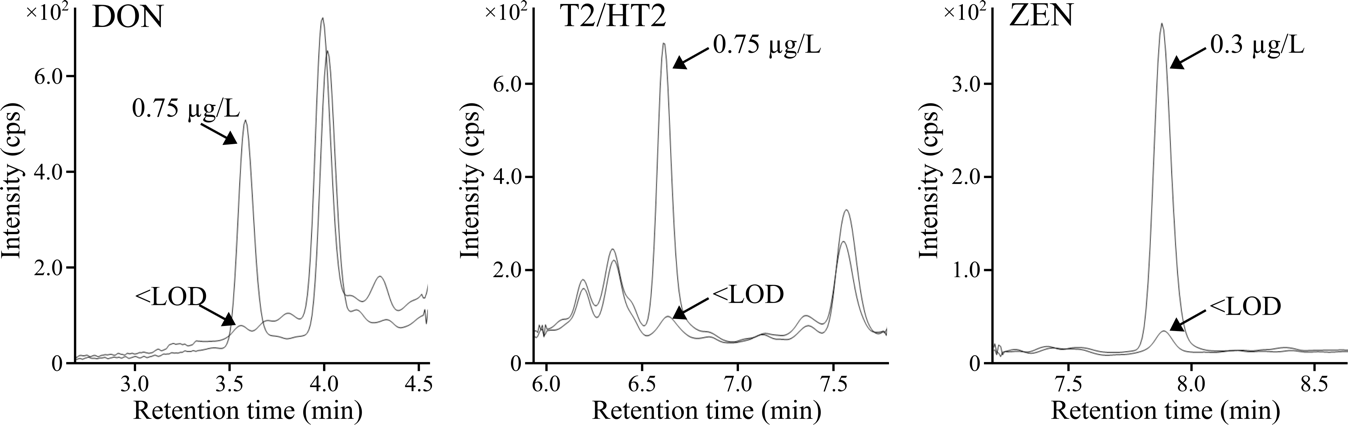

Supplement: Supplementary file 1 — Additional file 1: Table S1 Compound-specific mass spectrometer settings. Table S2 Characteristics of the participants obtained from self-administered questionnaire. Table S3 Total T2/HT2 concentrations in the present study and previous similar studies focusing on adult exposure. Table S4 ZEN concentrations in the present study and previous similar studies focusing on adult exposure. Fig. S1 Chemical structures of the mycotoxins measured in this study. Fig. S2 MRM chromatograms of mycotoxins in pooled human urine spiked with mycotoxins at two different concentrations of mycotoxins. [file ehpm-30-019-s001.docx]
